# Adipose Tissue Promotes a Serum Cytokine Profile Related to Lower Insulin Sensitivity after Chronic Central Leptin Infusion

**DOI:** 10.1371/journal.pone.0046893

**Published:** 2012-10-02

**Authors:** Emma Burgos-Ramos, Sandra Canelles, Arancha Perianes-Cachero, Eduardo Arilla-Ferreiro, Jesús Argente, Vicente Barrios

**Affiliations:** 1 Department of Endocrinology, Hospital Infantil Universitario Niño Jesús, Instituto de Investigación La Princesa and Department of Pediatrics, Universidad Autónoma de Madrid, Madrid, Spain; 2 Centro de Investigación Biomédica en Red Fisiopatología de Obesidad y Nutrición, Instituto de Salud Carlos III, Madrid, Spain; 3 Grupo de Neurobioquímica, Departamento de Bioquímica y Biología Molecular, Facultad de Medicina, Universidad de Alcalá, Alcalá de Henares, Spain; University of Las Palmas de Gran Canaria, Spain

## Abstract

Obesity is an inflammatory state characterized by an augment in circulating inflammatory factors. Leptin may modulate the synthesis of these factors by white adipose tissue decreasing insulin sensitivity. We have examined the effect of chronic central administration of leptin on circulating levels of cytokines and the possible relationship with cytokine expression and protein content as well as with leptin and insulin signaling in subcutaneous and visceral adipose tissues. In addition, we analyzed the possible correlation between circulating levels of cytokines and peripheral insulin resistance. We studied 18 male Wistar rats divided into controls (C), those treated *icv* for 14 days with a daily dose of 12 μg of leptin (L) and a pair-fed group (PF) that received the same food amount consumed by the leptin group. Serum leptin and insulin were measured by ELISA, mRNA levels of interferon-γ (IFN-γ), interleukin-2 (IL-2), IL-4, IL-6, IL-10 and tumor necrosis factor-α (TNF-α) by real time PCR and serum and adipose tissue levels of these cytokines by multiplexed bead immunoassay. Serum leptin, IL-2, IL-4, IFN-γ and HOMA-IR were increased in L and TNF-α was decreased in PF and L. Serum leptin and IL-2 levels correlate positively with HOMA-IR index and negatively with serum glucose levels during an *ip* insulin tolerance test. In L, an increase in mRNA levels of IL-2 was found in both adipose depots and IFN-γ only in visceral tissue. Activation of leptin signaling was increased and insulin signaling decreased in subcutaneous fat of L. In conclusion, leptin mediates the production of inflammatory cytokines by adipose tissue independent of its effects on food intake, decreasing insulin sensitivity.

## Introduction

Obesity is associated with an inflammatory state involved in the pathogenesis of many obesity related comorbidities. Previous findings indicate that inflammatory diseases mediate energy and weight deregulation though different proinflammatory cytokines [1], [2], whose levels are increased in both the circulation and peripheral tissues [3]. These changes predispose an individual to the development of type 2 diabetes mellitus, with this disease being associated with total and visceral obesity [4], [5].

Leptin modulates food intake, body weight and adipose stores, with a direct correlation between serum leptin levels, gene expression leptin in adipocytes and body fat [6]. Non-adipose cells are considered to be responsible for the production of the majority of proinflammatory factors [7], but adipocytes also synthetizes several cytokines [8]. Leptin also regulates immune function, playing a role in starvation-induced immunosuppression [9]. Deficient leptin signaling impairs cellular responses, whereas immune and malnutrition-related diseases are associated with increased synthesis of leptin and of inflammatory cytokines. In fact, leptin stimulates the production of proinflammatory cytokines by monocytes, largely distributed in the adipose tissue [10].

Hyperleptinemia is associated with insulin resistance. Although leptin initially increases insulin sensitivity, long-term exposure to high leptin levels has been reported to result in insulin resistance [11]. Leptin is a mediator of the inflammatory response that impairs insulin signaling in the hypothalamus and adipocytes [12], [13]. This inflammatory state favours the release of macrophage chemoattractant proteins, triggering insulin resistance that in turn induces a subsequent increase in circulating cytokines and fatty acids, leading to a lipotoxic state in non-adipose tissues that aggravates the pathological situation [14]. In addition, insulin resistance increases inflammatory cytokine synthesis in adipocytes, contributing to the exacerbation of this state [15].

The effect of exogenous leptin on insulin's actions and metabolic outputs has been studied mainly in leptin-deficient patients, as well as in models of experimental diabetes or obesity [11], [16]. However, there is little information in normal animals regarding the effect of leptin on the expression of proinflammatory cytokines in adipose tissue. The fact that leptin decreases food intake must also be kept in mind since the amount of food consumed may alter insulin sensitivity and the cytokine profile [17], [18], making it important to discriminate between the direct effects of leptin from those due to decreased food intake.

In the present study, we investigated how chronic exposure to increased leptin levels could modify the systemic cytokine profile and insulin resistance in a non-obese model. To discriminate between the direct effects of leptin and its induction of reduced food intake, a group of pair-fed rats was analyzed. The potential contribution of subcutaneous and visceral adipose tissues to the modifications in the cytokine profile was also examined.

## Results

### General characteristics of experimental groups

**Figure 1:**
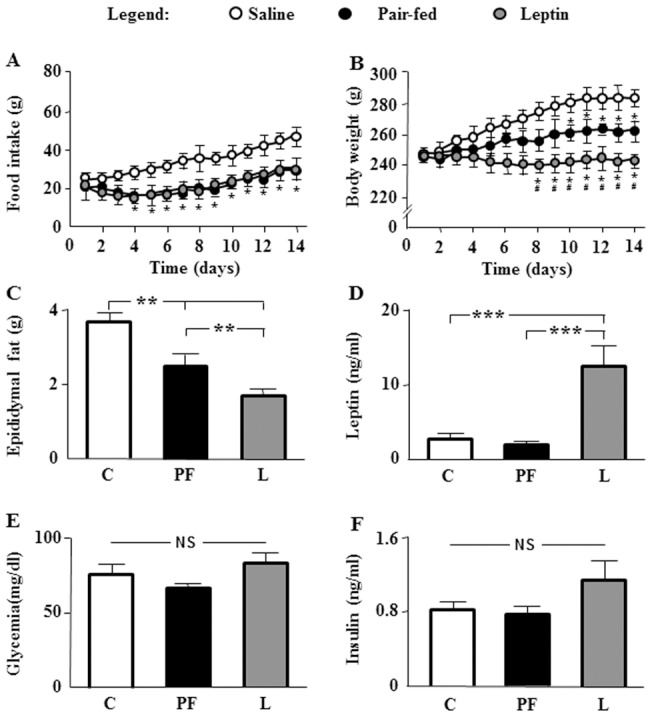
General characteristics of experimental animals. A. Mean daily food intake in rats that received saline (C) or chronic leptin (L) and the pair-fed group (PF). **B.** Mean body weight measurements throughout the study in the same groups. **C.** Epididymal fat content in the same groups. **D.** Serum leptin concentrations in the same groups. **E.** Serum glucose concentrations in the same groups. **F.** Serum insulin concentrations in the same groups. NS, non-significant; *p<0.05, **p<0.01, ***p<0.01 by ANOVA. * in panels A and B indicates significant difference (*p*<0.05) between C and L or PF groups, whereas ^#^ indicates significant difference (*p*<0.05) between PF and L groups.

Food intake and body weight were recorded to verify that *icv* leptin infusion affected these parameters. On the fourth day of treatment, food intake was reduced in L and PF with respect to C (Fig. 1A); whereas the appearance of differences in body weight in L was found on the eighth day with respect to the C and PF groups (Fig. 1B). Epididymal fat mass was reduced in both PF and L, with this reduction being greater in L (Fig. 1C). Serum leptin levels were increased in L (Fig. 1D). Glycemia (Fig. 1E) and serum insulin levels (Fig. 1F) showed no significant differences among the experimental groups.

### Chronic leptin administration changed serum proinflammatory cytokine levels, increased HOMA-IR index and attenuated the central and peripheral insulin effects on glycemia

**Figure 2:**
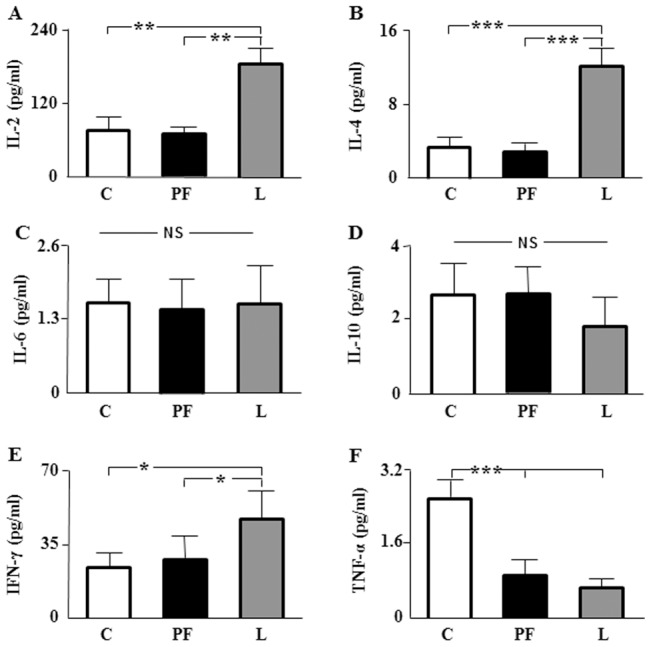
Serum levels of cytokines. A. Serum interleukin (IL)-2 levels in rats that received saline (C) or chronic leptin (L) and the pair-fed group (PF). **B.** Serum IL-4 levels in the same groups. **C.** Serum IL-6 levels in the same groups. **D.** Serum IL-10 levels in the same groups. **E.** Serum interferon γ (IFN-γ) levels in the same groups. **F.** Serum tumor necrosis α (TNF-α) levels in the same groups. NS, non-significant; *p<0.05, **p<0.01, ***p<0.001 by ANOVA.

Serum levels of IL-2, IL-4 and IFN-γ were increased in L with respect to C and PF (Fig. 2A, B and E, respectively). Interleukin-6 and -10 levels showed no differences among the groups (Fig. 2C and D, respectively), whereas TNF-α levels were reduced in PF and L (Fig. 2F).

**Figure 3:**
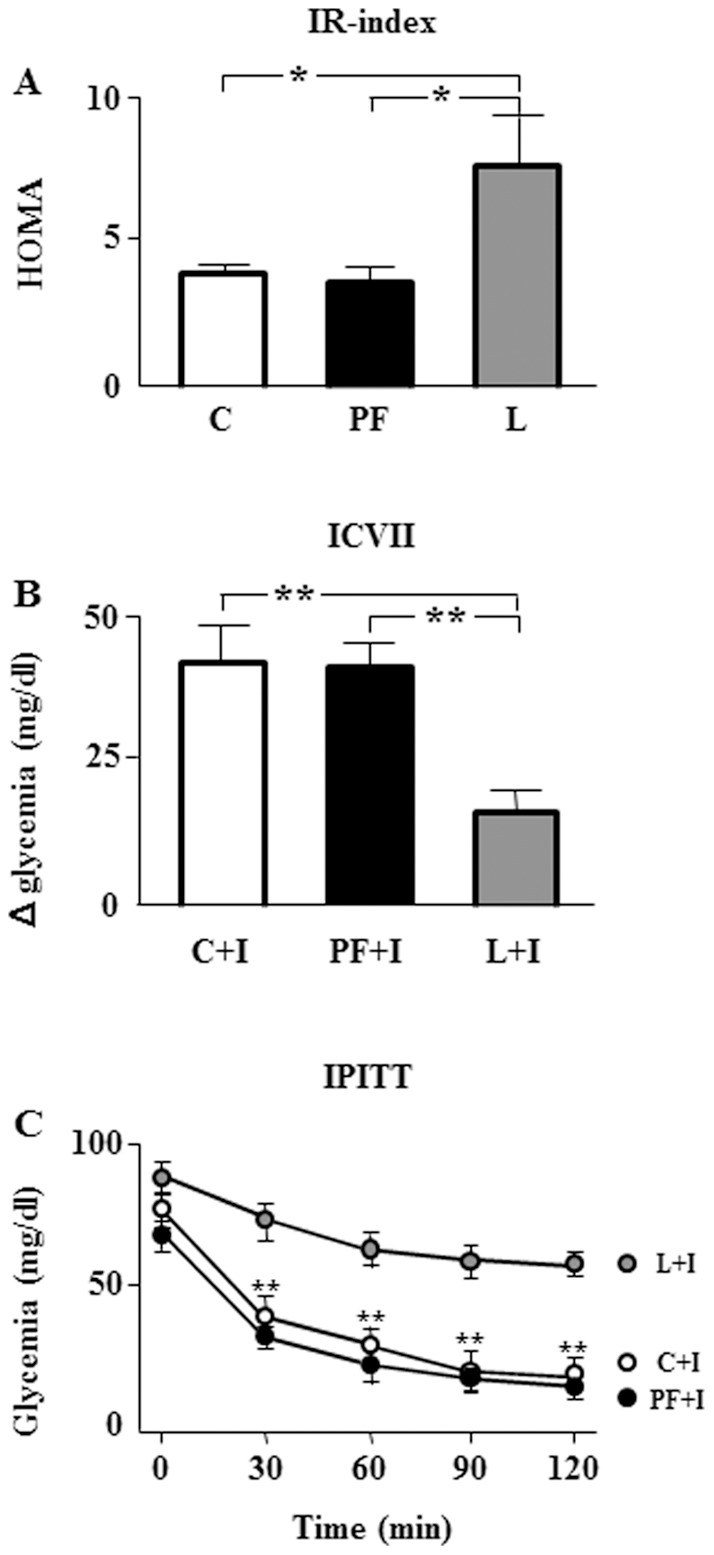
Homeostasis model assessment of insulin resistance (HOMA-IR), insulin sensitivity as delta (Δ) in glycemia after intracerebroventricular insulin infusion (ICVII) and glycemia during an intraperitoneal insulin tolerance test (IPITT). A. HOMA-IR index in rats that received saline (C) or chronic leptin (L) and pair-fed group (PF). **B.** Delta (Δ) in glycemia ([glycemia after 120 min of insulin bolus] – [glycemia before insulin bolus]) in rats that received saline plus acute insulin (insulin, C+I), chronic leptin plus acute insulin (L+I) and the pair-fed group plus insulin (PF+I). **C.** Serum glucose levels before (0 min) and during (30, 60, 90 and 120 min) an IPITT. *p<0.05, **p<0.01 by ANOVA.

As inflammation is correlated with insulin resistance [19] we first calculated the homeostasis model assessment of insulin resistance (HOMA-IR). This insulin-related resistance index was increased in L compared to C and PF (Fig. 3A). To examine if HOMA-IR shows a relationship with serum cytokine values, linear correlation regressions were performed. There was a positive correlation of HOMA-IR with serum leptin, IL-2 and IL-4 levels (Table 1).

**Table 1:** Linear correlations of homeostasis model assessment of insulin resistance (HOMA-IR), Δ glycemia ([glycemia after 120 min of insulin bolus] – [glycemia before insulin bolus]) after intracerebroventricular insulin infusion (ICVII) and area under the curve (AUC) after intraperitoneal insulin tolerance test (IPITT) with serum levels of leptin, interleukin (IL)-2 and IL-4.

	Leptin	IL-2	IL-4
HOMA-IR			
HOMA index	r = 0.64**	r = 0.66**	r = 0.67**
ICVII			
Δ glycemia	r = −0.66**	r = −0.64**	r = −0.57*
IPITT			
AUC	r = −0.58**	r = −0.56*	r = −0.39 (NS)

r, correlation coefficient; NS, non-significant; *p<0.05, **p<0.01.

As *icv* insulin administration increases glycemia [12], [20], we also evaluated insulin sensitivity by measuring changes in serum glucose levels after central insulin infusion. Although blood glucose levels were in the normal physiological range in all groups throughout the study, previous chronic exposure to leptin reduced the rise in glycemia induced by *icv* insulin injection (Fig. 3B). Negative correlations of delta of glycemia with serum leptin, IL-2 and IL-4 levels were found (Table 1).

As central leptin may modify peripheral insulin response, we also investigated whether chronic icv leptin infusion would be accompanied by reduced insulin tolerance. Although we found no differences in basal glycemia, a rapid drop of glycemia was observed throughout the ip insulin tolerance test (IPITT) in control and pair-fed rats, whereas only a modest reduction of glucose levels was detected in leptin-treated rats (Fig. 3C). Linear regression analyses showed that HOMA-IR presented a direct correlation with serum cytokine levels, whereas the correlation between the Δ in glycemia and cytokine levels was negative. Finally, the relationship of the area under the curve for glucose (AUC) after the IPITT was negative with leptin and IL-2 whereas no significant correlation with was observed with IL-4 levels (Table 1).

### Effect of leptin on relative mRNA and protein levels of cytokines in subcutaneous and visceral adipose tissues

**Figure 4:**
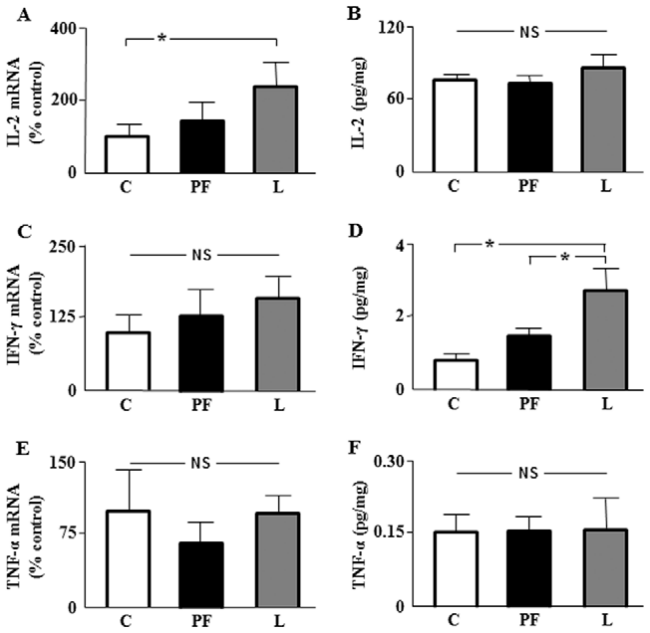
Relative mRNA and protein content of cytokines in subcutaneous adipose tissue. A. Relative mRNA levels of interleukin-2 (IL-2) in inguinal fat of rats that received saline (C) or chronic leptin (L) and pair-fed group (PF). **B.** Protein content of IL-2 in the same groups. **C.** Relative mRNA levels of interferon-γ (IFN-γ) in the same groups. **D.** Protein content of IFN-γ in the same groups. **E.** Relative mRNA levels of tumor necrosis factor α (TNF-α) in the same groups. **F.** Protein content of TNF-α in the same groups. NS, non-significant; *p<0.05 by ANOVA.

**Figure 5:**
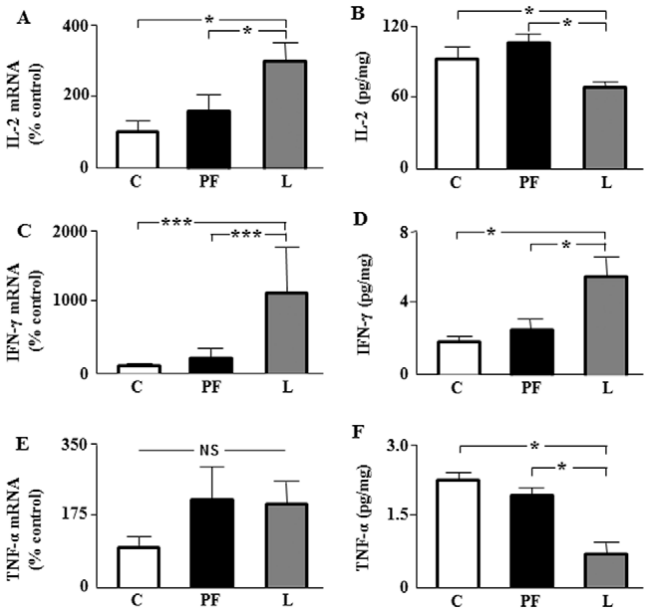
Relative mRNA and protein content of cytokines in visceral adipose tissue. A. Relative mRNA levels of interleukin-2 (IL-2) in epididymal fat of rats that received saline (C) or chronic leptin (L) and the pair-fed group (PF). **B.** Protein content of IL-2 in the same groups. **C.** Relative mRNA levels of interferon-γ (IFN-γ) in the same groups. **D.** Protein content of IFN-γ in the same groups. **E.** Relative mRNA levels of tumor necrosis factor α (TNF-α) in the same groups. **F.** Protein content of TNF-α in the same groups. NS, non-significant; *p<0.05, ***p<0.001 by ANOVA.

To determine whether adipose tissue contributes to the generation of the systemic inflammatory profile found in leptin-treated rats, we studied relative messenger RNA levels of IL-2, IL-4, IFN-γ TNF-α in subcutaneous and visceral adipose tissues. IL-2 mRNA levels were increased in subcutaneous and visceral tissues of L (Fig. 4A and 5A, respectively), whereas mRNA levels of IFN-γ only increase in visceral adipose tissue (Fig. 5C). No changes in the mRNA levels of TNF-α were seen in either tissue (Fig. 4E and 5E). Messenger RNA levels of IL-4 were very low and could not be adequately quantified.

Circulating cytokine levels are the result of the synthesis and liberation by several tissues. We analyzed protein concentrations of IL-2, IL-4, IFN-γ TNF-α in subcutaneous and visceral adipose tissue. IL-2 protein levels in subcutaneous adipose tissue were not different between the three groups (Fig. 4B), but was decreased in L with respect to C and PF in visceral adipose tissue (Fig. 5B). In L protein levels of IFN-γ were increased in both adipose depots (Fig. 4D and 5D) and TNF-α decreased in visceral adipose tissue (Fig. 5F), with no change in subcutaneous fat (Fig. 4F, Table 1). Finally, protein concentrations of IL-4 were increased in the subcutaneous adipose tissue of L with respect to C and PF groups (6.12±0.87, 5.76±1.09 and 8.94±0.61, expressed in pg of IL-4/mg of protein in C, PF and L groups, respectively), whereas no changes in visceral adipose tissue were seen (11.44±2.37, 10.44±1.06 and 10.54±0.92, expressed in pg of IL-4/mg of protein in C, PF and L groups, respectively).

### Immune and inflammatory markers in the different adipose compartments

**Figure 6:**
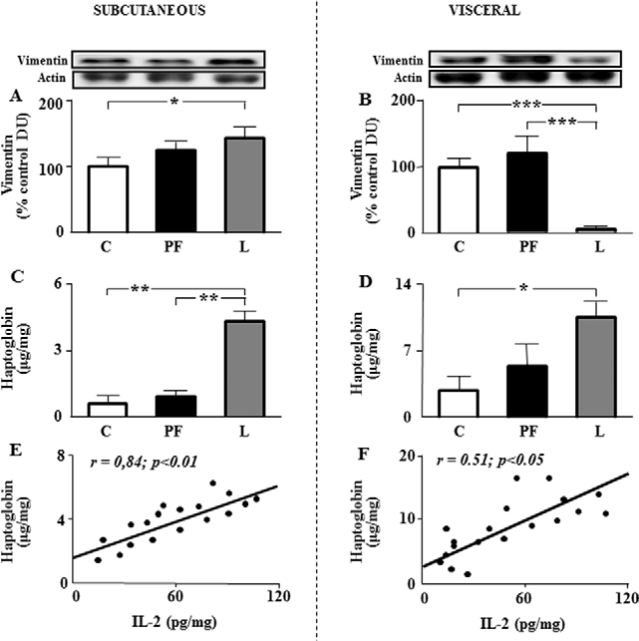
Immune and inflammatory markers in adipose compartments. A. Relative vimentin protein levels in inguinal fat of rats that received saline (C) or chronic leptin (L) and the pair-fed group (PF). **B.** Relative vimentin protein levels in epididymal fat of the same groups. **C.** Haptoglobin levels in inguinal fat of the same groups. **D.** Haptoglobin levels in epididymal fat of the same groups. **E.** Linear regression analysis between interleukin-2 (IL-2) and haptoglobin in inguinal fat. **F.** Linear regression analysis between interleukin-2 (IL-2) and haptoglobin in epididymal fat. Correlation coefficients (*r*) and *p* values are represented for each analysis. DU, densitometry units; *p<0.05 by ANOVA, ***p<0.001 by ANOVA.

As specific cell infiltration could affect the reported inflammatory profile, we have analyzed several markers, expressed in different cell types. The levels of F4/80 are undetectable in subcutaneous and visceral adipose tissue of controls; however, a strong signal was detected in both fat depots of L group. Vimentin was increased in subcutaneous compartment of L (Fig. 6A) and decreased in visceral adipose tissue of L group (6B). Finally, levels of haptoglobin were increased in subcutaneous (Fig. 6C) and visceral adipose tissue (Fig. 6D), showing a direct correlation with IL-2 in both localizations (Fig. 6E and 6F).

### Intracellular signaling pathways of leptin and insulin in adipose tissue after leptin and insulin infusion

**Figure 7:**
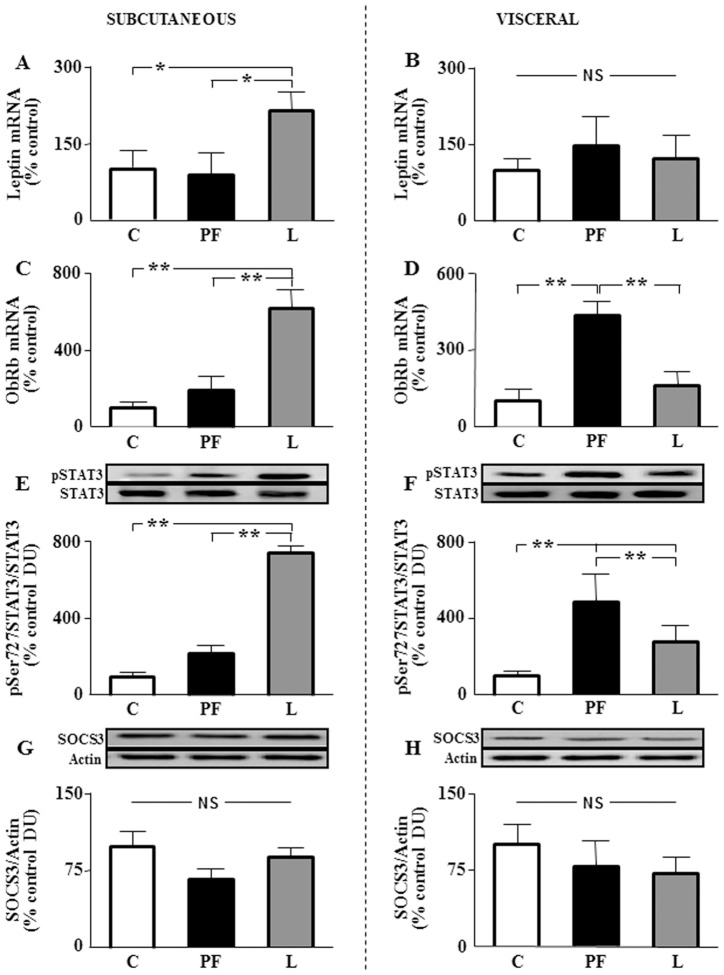
Leptin signaling in subcutaneous and visceral fat. A. Relative mRNA levels of leptin in inguinal fat of rats that received saline (C) or chronic leptin (L) and the pair-fed group (PF). **B.** Relative mRNA levels of leptin in epididymal fat of the same groups. **C.** Relative mRNA levels of the long form of the leptin receptor (ObRb) in inguinal fat of the same groups. **D.** Relative mRNA levels of ObRb in epididymal fat of the same groups. **E.** Relative phosphorylated (p) signal transducer and activator of transcription factor 3 phosphorylated on serine 727 (pSer727-STAT3) protein levels in inguinal fat of the same groups. **F.** Relative pSer727-STAT3 protein levels in epididymal fat of the same groups. **G.** Relative suppressor of cytokine signaling 3 (SOCS3) protein levels in inguinal fat of the same groups. **H.** Relative SOCS3 protein levels in epididymal fat of the same groups. The data are expressed as a percentage of the control ratio. DU, densitometry units; NS, non-significant; *p<0.05 by ANOVA **p<0.01 by ANOVA.

Leptin mRNA levels were increased in subcutaneous fat of L (Fig. 7A), with no change in visceral fat (Fig. 7B). The mRNA levels of ObRb were increased in subcutaneous adipose of L (Fig. 7C) and in visceral fat of PF (Fig. 7D). Phosphorylation of signal transducer and activator of transcription 3 (STAT3) at Ser727 was increased in the subcutaneous adipose tissue of L (Fig. 7E), whereas in visceral tissue it was increased in both PF and L, with this increase being greater in PF (Fig. 7F). Levels of suppressor of cytokine signaling 3 (SOCS3) were unchanged in subcutaneous and visceral fat (Fig. 7G and 7H, respectively and Table 2).

**Table 2:** Serum cytokine levels (pg/ml), insulin resistance or sensitivity indexes, relative mRNA levels and concentration of cytokines in subcutaneous and visceral adipose tissue (pg/mg of protein) and intracellular signaling (expressed as % control) in both fat depots.

			Control	Pair-fed	Leptin
Serum	Cytokines	Leptin	3.04±0.63	2.02±0.52	13.75±2.86 *** ^###^
		IL-2	75.5±22.1	70.6±9.8	185.7±23.6 ** ^##^
		IL-4	3.39±1.16	2.81±0.88	12.07±2.00 *** ^###^
		IFN-γ	24.4±6.8	28.4±10.3 *	44.8±12.8 * ^#^
		TNF-α	2.61±0.37	0.95±0.32 ***	0.67±0.22 ***
	Insulin- indexes	HOMA-IR	3.98±0.45	3.70±0.52	7.72±1.76 * ^#^
		Δ glycemia	42.9±5.4	40.7±3.8	18.5±4.0 ** ^##^
Subcutaneous/ visceral	Relative mRNA levels	IL-2	100±34/ 100±32	143±55/ 161±48	239±66 */ 303±56 *
		IFN-γ	100±29/ 100±31	131±46/ 223±112	159±37/ 1126±648 *** ^###^
		TNF-α	100±42/ 100±25	67±21/ 216±80	98±19/ 208±54
	Protein levels	IL-2	76.0±4.7/ 92.5±9.6	73.8±5.5/ 102.8±7.0	86.3±10.8/ 68.5±5.9 * ^#^
		IFN-γ	0.82±0.20/ 1.84±0.34	1.14±0.22/ 2.60±0.52	2.76±0.62 * ^#^/ 5.58±1.05 * ^#^
		TNF-α	0.15±0.03/ 2.29±0.15	0.14±0.02/ 1.96±0.14	0.16±0.06/ 0.71±0.24 * ^#^
	Immune – inflammatory markers	Vimentin	100±15/ 100±13	124±13/ 119±26	144±15 */ 5±1 *** ^###^
		Haptoglobin	1.01±0.59/ 2.83±1.53	1.56±0.26/ 5.46±2.27	7.16±0.69 ** ^##^/ 10.60±1.54 *
	Intracellular signaling	ObRb	100±18/ 100±39	198±69/ 439±49 **	620±92 ** ^##^/ 163±52 ^##^
		p/t STAT3	100±22/ 100±33	258±23/ 508±139 **	744±57 ** ^##^/ 242±76 ** ^##^
		SOCS3	100±13/ 100±19	66±9 79±24	88±9/ 72±16
		IR	100±13/ 100±11	121±10/ 95±18	130±9/ 59±22 * ^#^
		p/t Akt	100±12/ 100±1	47±21 */ 58±7 *	42±19 */ 27±4 * ^#^
		FOXO1	100±24/ 100±17	136±33/ 127±13	207±40 */ 182±36 * ^#^
		PTP1B	100±25/ 100±9	109±28/ 109±21	187±11 * ^#^/ 14±8 *** ^###^

FOXO1, forkhead box O number 1; HOMA-IR, homeostasis model assessment of insulin resistance; IL, interleukin; IFN-γ, interferon-γ; IR, insulin receptor; ObRb, long form of leptin receptor; p/t, phosphorylated/total; PTP1B, protein tyrosine phosphatase 1B; SOCS3, suppressor of cytokine signaling 3; STAT3, transducer and activator of transcription factor 3; TNF-α, tumor necrosis factor α. *p<0.05, **p<0.01, ***p<0.001 by ANOVA vs. control; ^#^ *p*<0.05, ^##^ *p*<0.01, ^###^ *p*<0.001 by ANOVA vs. pair-fed group.

**Figure 8:**
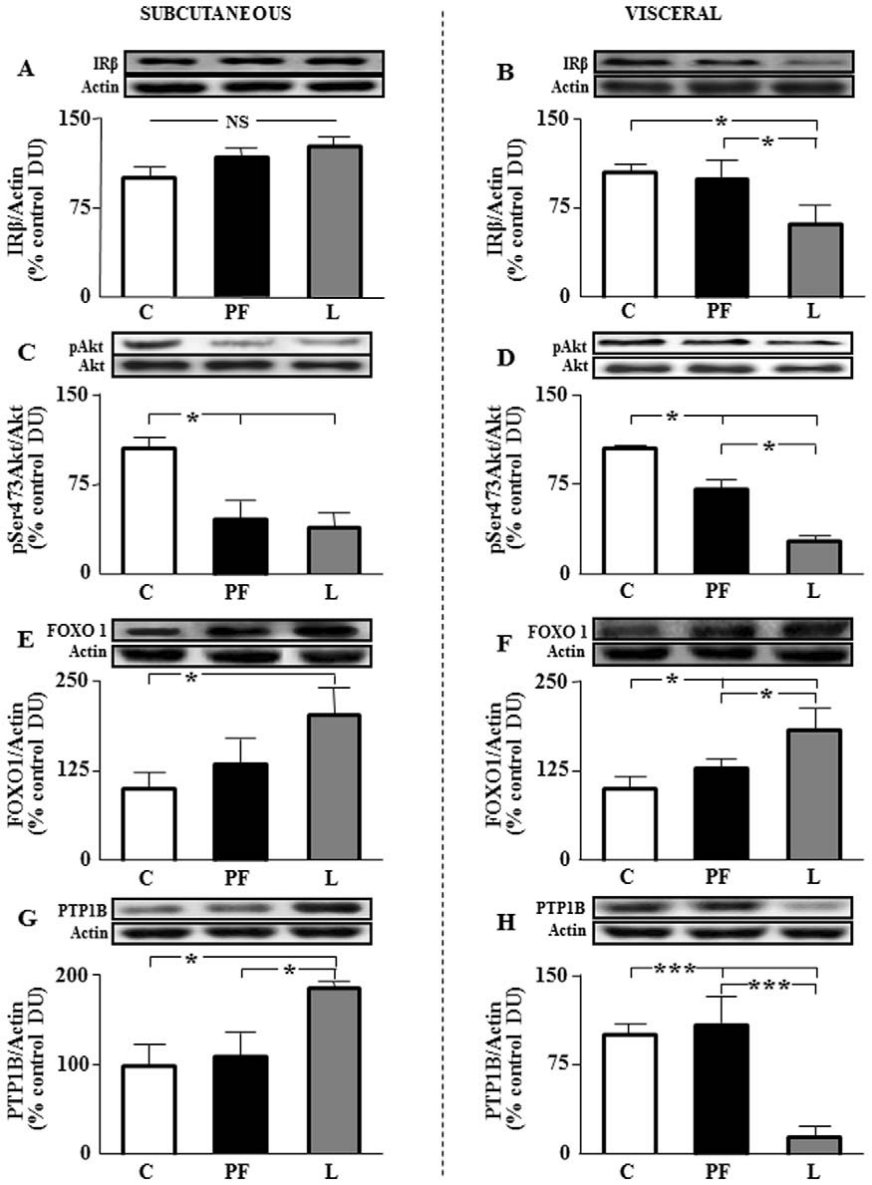
Insulin signaling in subcutaneous and visceral fat. A. Relative levels of the insulin receptor beta chain (IRβ) in inguinal fat of rats that received saline (C) or chronic leptin (L) and the pair-fed group (PF). **B.** Relative IRβ levels in epididymal fat of the same groups. **C.** Relative phosphorylated (p) Akt on serine 473 (pSer473-Akt) protein levels in inguinal fat of the same groups. **D.** Relative pSer473-Akt protein levels in epididymal fat of the same groups. **E.** Relative levels of the forkhead box-containing protein O-1 (FOXO1) in inguinal fat of the same groups. **F.** Relative FOXO1 levels in epididymal fat of the same groups. **G.** Relative levels of the protein tyrosine phosphatase 1B (PTP1B) in inguinal fat of the same groups. **H.** Relative PTP1B levels in epididymal fat of the same groups. The data are expressed as a percentage of the control ratio. DU, densitometry units; NS, non-significant; *p<0.05 by ANOVA, ***p<0.001 by ANOVA.

To determine if chronic exposure to increased leptin levels modulates insulin signaling, we examined insulin receptor β chain (IRβ) levels in subcutaneous and visceral tissues. Levels of IRβ in subcutaneous fat were not different between the experimental groups (Fig. 8A) and were reduced in visceral fat of L with respect to C and PF (Fig. 8B). Phosphorylation of Akt at Ser 473 was reduced in subcutaneous and visceral adipose of PF and L (Fig. 8C and 8D, respectively). No differences were detected in phosphorylation of this target between PF and L in subcutaneous tissue (Fig. 8C), whereas in visceral adipose tissue, the levels of Akt phosphorylation were lower in L compared to PF (Fig. 8D).

We have determined Akt activation after insulin infusion in both fat pads. Phosphorylation of Akt at Ser 473 was reduced in subcutaneous adipose tissue of leptin-treated rats (100.0±7.9 vs. 44.8±4.1, p<0.01; in C plus insulin and L plus insulin, respectively), whereas no differences were detected in visceral fat (100.0±20.2 vs. 119.3±14.7, p = 0.79; in C plus insulin and L plus insulin, respectively) after central insulin infusion. We also analyzed Akt activation after IPIIT in both compartments. Phosphorylation of Akt at Ser 473 was decreased in subcutaneous adipose tissue (100.0±9.7 vs. 57.0±6.5, p<0.05; in C plus insulin and L plus insulin, respectively) as in visceral fat (100.0±11.2 vs. 75.6±3.4, p<0.05; in C plus insulin and L plus insulin, respectively).

Levels of forkhead box-containing protein O-1 (FOXO1) were increased in subcutaneous fat of L and visceral fat of PF and L (Fig. 8E and 8F, respectively, Table 2). Protein tyrosine phosphatase 1B (PTP1B) was increased in L respect to C and PF in subcutaneous adipose tissue (8G) and diminished in visceral depot of L group (8H).

## Discussion

This study demonstrates that chronic central leptin administration causes a serum inflammatory profile correlated to insulin resistance. Adipose tissue contributes to the rise in circulating cytokine levels in leptin-treated rats and this increase does not seem to be correlated to the lower insulin sensitivity in adipose tissue. A direct effect of increased leptin levels exists as serum levels of most cytokines did not differ between pair-fed rats and controls.

Leptin induced a proinflammatory profile in serum, as previously shown [21], consistent with an elevation of inflammatory cytokines and leptin in obese patients [22]. There is increasing evidence that leptin enhances proinflammatory immune responses in different blood cell types, some of which are infiltrated in adipose tissue [23]. In addition, correlations between serum levels of leptin and several interleukins, as well as with IFN-γ, have been shown in several pathologies associated with inflammation [24], [25], [26]. In spite of an increase in several proinflammatory cytokines, serum TNF-α levels were reduced after leptin infusion. However, this decline is most likely not a direct effect of leptin, as similar TNF-α level were found in pair-fed rats. In fact, reduced food intake may have some anti-inflammatory effects and a low caloric diet not only ameliorates serum proinflammatory profiles, but also decreases the mRNA expression of up-regulated chemokines in obese patients [27].

Our experimental model reproduces a low-grade peripheral inflammatory situation without obesity, but where hyperleptinemia is present. We and others have reported that this experimental model of leptin infusion induces weight loss, central changes in leptin and insulin signaling and hyperleptinemia [12], [28]. Although we cannot discard the contribution of exogenous leptin to the rise in serum levels, leptin synthesis in subcutaneous adipose tissue is augmented and this may contribute to the increase in circulating levels. The mechanism by which an increase in central leptin levels stimulates leptin expression in fat depots is not well known, but it has been reported that central leptin infusion stimulates triiodothyronine production [29], [30]. This thyroid hormone increases the expression of leptin mRNA in subcutaneous fat depots [31] and also in adipocytes *in vitro* [32]. Although the main role of leptin is to regulation body weight by affecting food intake [33], it has additional effects on carbohydrate and lipid metabolism [34] that could explain the differences in weight gain between pair-fed and leptin-treated rats. Additionally, weight loss could also be potentiated by the levels of systemic cytokines [35].

Previous studies have demonstrated the relationship between obesity, hyperleptinemia and reduced insulin sensitivity and the beneficial effect of weight loss on insulin action [36], [37]. Our model produces a low-grade peripheral inflammation without obesity where hyperleptinemia is present and there is a correlation between leptin and several interleukins with peripheral insulin resistance. This interesting finding has been previously reported in patients where plasma leptin levels correlate with HOMA-IR independently of the effect of obesity [38], indicating a key role of hyperleptinemia *per se* in the generation of this adverse profile and suggesting that the contribution of adipose tissue may be more closely related to its functional state, than to its total amount. In addition, a direct effect of leptin has been shown in clinical studies where induced hyperleptinemia contributes to increase insulin resistance, whereas interruption of treatment ameliorates the sensitivity to insulin [11]. We have assessed insulin sensitivity after insulin challenge by using a central insulin bolus, because this infusion increased glycemia [12], [39]. The attenuated response in L suggest that chronic exposure to *icv* leptin decreased central insulin control of glucose levels [20], although we cannot exclude an effect of leptin treatment on peripheral tissues. Finally, we also evaluated insulin sensitivity after a classical intraperitoneal insulin tolerance test, observing a lower decrease in serum glucose levels in leptin-treated rats, thus suggesting reduced insulin sensitivity. Taken together, these results seem to indicate that leptin modify the response to insulin administration by decreasing its sensitivity.

Two of the main targets of leptin are STAT3 and SOCS3. In adipose tissue activation of STAT3 was coincident with increased expression of the leptin receptor. Although the direct action of leptin on the expression of its receptor is controversial, a positive effect has been previously reported [40], as we described here in inguinal adipose tissue. In contrast to subcutaneous adipose, in epididymal fat leptin receptor mRNA levels were increased in pair-fed rats. The positive effect of caloric restriction on leptin receptors has been reported [41], suggesting that moderate food reduction improves leptin signaling in some localizations. Hence, depot-specific differences have been reported both in experimental animals [42] and lean and obese patients [43].

Leptin modulates the production of interleukins in different cell types, including T cells and macrophages [44], [45] and generates an inflamed state in adipose tissue, probably regulated by crosstalk between adipocytes and macrophages [46], [47]. As mentioned above, the main findings reported here are the increase in mRNA levels of IL-2 in both inguinal and epididymal fat pads, the marked augment in IFN-γ mRNA in epididymal fat, and particularly, the increased pSTAT3 levels without changes in SOCS3 in subcutanteous fat in response to chronic leptin administration. The lack of leptin-induced STAT3 signaling is associated with reduced cytokine production [48], whereas leptin exposure increases both STAT3 activation and chemokine expression in macrophages [10]. Thus, the activation of leptin signaling reported here may account for the rise in the mRNA levels of cytokines. Not all changes in mRNA levels were related with changes in protein levels in adipose tissue, which is most likely due to increased secretion that would contribute to the generation of the serum inflammatory profile. However, the relationship is direct for some cytokines, such as IFNγ in visceral adipose. In contrast, TNF-α gene expression and levels in adipose tissue do not show an apparent relationship with serum profile. We must keep in mind that reduced food intake reduces white adipose tissue and leptin selectively decreases visceral adiposity [49], as we report here. Thus, although there are no changes in TNF-α mRNA levels per gram of adipose tissue, a decrease in the total amount of adipose tissue could contribute to the reduction of serum TNF-α.

Obesity and hyperleptinemia are associated with a proinflammatory state, with infiltration of different cell types, such as macrophages into adipose tissue [7]. The presence of the macrophage marker F4/80 after chronic leptin infusion indicates the presence of these cells in both fat depots that could participate in several inflammatory pathways [50]. Moreover, an interaction between macrophages and adipocytes has been reported [51], affecting insulin resistance mediated by the reduction of Akt phosphorylation, as we show here. Mesenchymal stromal cells, present in adipose tissue, are involved in anti-inflammatory processes, modulating the secretion of several interleukins [52]. We have found a reduction of vimentin, a mesenchymal marker [53], [54] in visceral fat depot, which could exacerbate inflammation and partial insulin resistance observed after chronic leptin treatment. In this regard, transplantation of adipose-derived mesenchymal cells increased phosphorylation of Akt [55], whereas macrophages inhibit differentiation of these cells. Hence, infiltration of macrophages in patients with obesity restrains this physiological process via secretion of proinflammatory cytokines [56]. Adipocytes also participate in the inflammatory state, expressing cytokines and their receptors [8]. Haptoglobin, a marker of inflammation is mainly synthetized by hepatocytes and adipocytes and its synthesis is upregulated by cytokines [57]. This adipose marker could exacerbate insulin resistance as it has been reported than impairment of glucose homeostasis is diminished by haptoglobin deficiency [58].

The data reported herein indicate that food restriction impairs insulin signal transduction in subcutaneous and visceral fat pads with leptin potentiating this effect in visceral tissue. Although reduction of food intake may initially attenuate insulin resistance [59], it has been stated that food restriction during the same length of time could lead to a down-regulation of insulin signaling in adipose tissue [60]. These authors demonstrate that this negative effect was due to the reduction in phosphorylation of the insulin receptor, together with a decrease of insulin receptor substrates, which could explain the reduced Akt phosphorylation. It appears that decreased insulin sensitivity in adipose tissue does not contribute in a significant manner to the change in the proinflammatory profile as pair-fed rats exhibit a cytokine profile similar to controls, with the exception of the levels of TNF-α. However, the pronounced reduction of Akt phosphorylation in visceral fat in the L group could be related to a direct effect of leptin and/or cytokines, as leptin directly decreases insulin-dependent autophosphorylation in adipocytes [13] and inflammatory cytokines may induce insulin resistance by decreasing insulin receptor levels [61], as we found in visceral fat. Alternatively, cytokine down-regulation of insulin receptor substrate expression [62] could explain the profound reduction of Akt phosphorylation reported here. Chronic leptin-treated rats present higher FOXO1 levels that could be the result of the lower Akt phosphorylation. In fact, activation of Akt reduces this factor by promoting subsequent polyubiquitylation and degradation by an ubiquitin proteasome system [63]. Thus, higher hypothalamic levels of FOXO1 in L group could be the result of a leptin-induced reduction in nuclear export of FOXO1 and cytoplasmic degradation [64]. Protein tyrosine phosphatase1B has been shown to be a negative regulator of insulin action and we have found an increase of PTP1B beside a reduction of insulin signaling in subcutaneous fat depot, as it has previously reported [65]. Unexpectedly, we observed a great reduction of levels of this protein in visceral fat. Nevertheless, it has been recently shown that PTP1B deficiency can exacerbate inflammatory processes [66], showing higher sensitivity to IFN-γ effects [67].

We cannot discard a negative effect of IFN-γ on insulin sensitivity in adipose tissue, as its administration induces sustained loss of insulin-stimulated glucose uptake, coincident with reduced Akt phosphorylation and STAT activation [61]. These authors also observed that JAK inhibition restored glucose uptake and Akt phosphorylation, in accordance with the results reported here showing an inverse relationship between IFN-γ levels and insulin signaling. In a similar way, increased serum IL-2 levels, as well as synthesis of IL-2 in epididymal fat, may contribute to reduce insulin sensitivity in adipose tissue [8]. Thus, these results suggest that leptin could affect insulin signaling in visceral tissue by modulating the synthesis of cytokines.

Several caveats should be taken into consideration when evaluating these results. Adipose tissue has different cell types and this study does not quantify the contribution of adipocytes or other cells in the generation of an inflammatory response. We also must take into account that we have reported a decrease in the content of epididymal fat, but not in subcutaneous adipose tissue, which was not been quantified. However, previous studies indicate that food restriction and leptin administration at similar doses decrease subcutaneous fat content by approximately 50% [68], [69]; thus, the contribution of fat depots to circulating cytokine levels is the resultant of the balance between cytokine synthesis and amount of adipose tissue. We cannot differentiate between the central effects of leptin from those due to increased circulating levels, as changes in metabolism and gene expression in fat are similar in most of the analyzed parameters by both administration routes [32] and denervation of white adipose tissue is necessary to make this distinction. In addition, synthesis of leptin in adipose tissue also may contribute to increased serum levels. Central infusion was chosen as it has more profound effects not only in food restriction and body weight, but also on carbohydrate and lipid metabolism [70], [71]. Finally, although we did not detect seizures or coma after i.p. insulin administration, the possible appearance of these symptoms in fasted animals should be taken into account. In addition, the fact that counterregulatory mechanisms in fasted animals will be activated in an attempt to avoid the hypoglycemia and this response could be different between the experimental groups must be considered. To avoid this marked decrease in serum glucose levels after the IPITT, a lower dose of insulin [72] could possibly be used.

In conclusion, our findings indicate that chronic leptin administration produces a serum inflammatory profile closely correlated with lower insulin sensitivity. Adipose tissue contributes to the generation of this adverse profile and the increased synthesis of cytokines seems to be related with the activation of leptin signaling and independent of insulin resistance in both fat depots. These results suggest that pharmacological inhibition of leptin signaling in adipose tissue could be of interest for reducing the low grade inflammatory state associated to obesity.

## Materials and Methods

### Materials

All chemicals were purchased from Sigma-Aldrich, Inc. (St. Louis, MO, USA) unless otherwise noted. The Inmmun-Star Western C Kit (ECL) was from Bio-Rad Laboratories (Hercules, CA, USA) and recombinant rat leptin was purchased from Preprotech (Rocky Hill, NJ, USA). Antibodies for phosphorylated (p)-Ser473-Akt, p-Ser727-signal transducer and activator of transcription factor 3 (pSer727-STAT3) and suppressor of cytokine signaling 3 (SOCS3) were from Cell Signaling Technology (Danvers, MA, USA), antibodies against Akt, F4/80, FOXO1 and IRβ were from Santa Cruz Biotechnology Inc. (Santa Cruz, CA, USA), the antibody against STAT3 from R&D Systems (Minneapolis, MN, USA) and the antibody to actin from Thermo Fisher Scientific (Fremont, CA, USA). Antibody for PTP1B was from Millipore Corporate Headquarters (Billerica, MA, USA) and antibody against vimentin from Sigma-Aldrich. The corresponding secondary antibodies conjugated with horseradish-peroxidase were purchased from Thermo Fisher Scientific Inc. (Waltham, MA, USA).

The rat leptin and insulin ELISA kits and multiplexed bead immunoassay for IL-2, -4, -6 -10, IFN-γ and TNF-α were from Millipore Corporate Headquarters (Billerica, MA, USA). TaqMan gene expression assays were purchased from Applied Biosystems (Foster City, CA, USA).

### Animals

This study was approved by the Ethics Committee of the Universidad de Alcalá de Henares (SAF 2010–22277, Ministerio de Ciencia y Tecnología) and complied with Royal Decree 1201/2005 (Boletín Oficial del Estado, BOE no. 252) pertaining to the protection of experimental animals and with the European Communities Council Directive (86/609/EEC).

Thirty-six adult male Wistar rats (250±10 g) were individually caged with a 12-h light/dark cycle and fed standard chow and water *ad libitum*. After an overnight fast, rats were anesthetized (0.02 ml of ketamine/100 g wt and 0.04 ml of xylazine/100 g wt) and positioned in a stereotaxic apparatus. A cannula attached to an osmotic minipump (Alzet, Durect Corp., Cupertino, CA, USA) containing either saline or leptin was implanted into the right cerebral ventricle (−0.3 mm anteroposterior, 1.1 mm lateral from Bregma). Leptin was dissolved in saline plus 1% BSA and insulin was dissolved in PBS. Rats were treated *icv* for 14 days with either saline with 1% BSA or leptin (12 μg/day). To discriminate between the direct effects of leptin from those due to induction of decreased food intake, we included a pair-fed group that received the same amount of food consumed by the leptin-treated group the day before. Food intake and body weight were measured daily. Rats were sacrificed by decapitation at 8.00 h after a 12 h fast, inguinal fat as subcutaneous adipose tissue and epididymal fat as visceral tissue were isolated and blood collected.

### Determination of insulin sensitivity, insulin tolerance and insulin resistance

On the last day of infusion, after a fasting period of 12 hours, 10 mIU of insulin or PBS in a volume of 5 μl were injected *icv* and rats were sacrificed by decapitation 2 hours later. This resulted in the following groups (n = 6 per group): chronic saline with 1% BSA (control, C), chronic saline with 1% BSA plus caloric restriction (pair-fed, PF), chronic leptin (leptin, L), chronic saline with 1% BSA plus acute insulin (insulin, I), 5) chronic saline with 1% BSA plus acute insulin and caloric restriction (insulin + pair-fed, PF+I) and 6) chronic leptin plus acute insulin (leptin + insulin, L+I). Blood was incubated at room temperature for 30 min, centrifuged at 1,500 *g* for 10 min at 4°C and serum collected and frozen at −80°C until determination of leptin, insulin and cytokines. Glycemia was measured before and 120 min after insulin administration via tail puncture (Accu-Check Sensor, Roche, Mannheim, Germany).

Insulin tolerance was assessed by performing an *ip* insulin tolerance test (IPITT) after the injection of a bolus of insulin (Regular Humuline, Lilly; 2 U/kg *ip*) and blood samples were drawn consecutively at 0, 30, 60, 90 and 120 min for glucose measurement [73], as described above.

In addition, homeostasis model assessment of insulin resistance (HOMA-IR) index was calculated with the following formula: HOMA-IR  =  [Glucose (mmol/l) × insulin (μU/ml)]/22.5. Insulin sensitivity was measured as delta (Δ) in glycemia, calculated as glycemia after 120 min of insulin bolus minus glycemia before insulin bolus.

### ELISAs

Serum leptin and insulin levels were measured with ELISA kits according to the manufacturer's instructions. The sensitivity of the assays for leptin and insulin were 0.04 and 0.2 ng/ml, respectively. The intra-assay variations were 2.2% for leptin and 1.9% for insulin and the inter-assay variations were 3.4% for leptin and 7.6% for insulin. Haptoglobin concentrations in adipose fat depots were determined by an ELISA kit from AssayPro (St. Charles, MO, USA). The intra- and inter-assay variations for haptoglobin were 5.2% and 7.9%, respectively.

### Tissue homogenization and protein quantification

Adipose tissues were homogenized on ice in 500 μl radioimmunoprecipitation assay lysis buffer (RIPA; 50 mM NaH_2_PO_4_, 100 mM Na_2_HPO_4_, 0.1% sodium dodecyl sulfate, 0.5% NaCl, 1% Triton X-100) with EDTA-free protease inhibitors (Roche Diagnostics, Barcelona, Spain), 1 mM phenylmethanesulfonylfluoride and 5 mg/ml sodium deoxycholate for extraction of cytokines and leptin and insulin signaling-related proteins. The lysates were incubated overnight at −70°C and then centrifuged at 12,000 *g* for 5 min at 4°C. The supernatant was stored at −80°C until assayed. Total protein concentration was determined by the method of Bradford (Bio-Rad Laboratories).

### Multiplexed bead immunoassay

Serum and tissue IL-2, -4, -6, -10, IFN-γ and TNF-α concentrations were measured by a multiplexed bead immunoassay. Briefly, after blockage of the filter plate with assay buffer, wells were washed by using a vacuum manifold and beads with different fluorescent labeling for each antigen conjugated to the appropriate antibodies and serum (25 μl each) were added and then incubated for 18 hours at 4°C. Wells were washed and 25 μl of antibody conjugated to biotin were added. After incubation for 30 min at room temperature, beads were incubated during 30 min with 50 μl streptavidin conjugated to phycoerythrin. After washing, beads were resuspended and a minimum of 50 beads per parameter was analyzed in the Bio-Plex suspension array system 200 (Bio-Rad Laboratories, Madrid, Spain). Raw data (mean fluorescence intensity, MFI) was analyzed by using the Bio-Plex Manager Software 4.1 (Bio-Rad Laboratories). Sensitivity is approximately 2–5 pg/ml, mean intra-assay variation was 8.0% and mean inter-assay variation was 12.6% for all cytokines.

### Western blotting

Western blotting was used to determine levels of SOCS3 and insulin receptor and activation of STAT3 and Akt in subcutaneous and visceral adipose tissues. The proteins were resolved on a 10% SDS-polyacrylamide gel and then transferred to polyvinyl difluoride (PVDF) membranes. Membranes were blocked with TTBS containing 5% (w/v) BSA during 2 h at 25°C and incubated with the corresponding primary antibody (diluted 1∶1000) in TTBS at 4°C overnight. The membranes were subsequently washed and incubated with the corresponding secondary antibody conjugated with peroxidase at a dilution of 1∶2000 in TTBS during 90 min at 25°C. The proteins were detected by chemiluminescence with an ECL system. Quantification of the bands obtained was carried out by densitometry using a Kodak Gel Logic 1500 Image Analysis system and Molecular Imaging Software version 4.0 (Rochester, NY, USA). Insulin receptor, FOXO1, PTP1B, SOCS3 and vimentin were normalized with actin and pAkt and pSer727STAT3 with their total forms.

### RNA purification and real time PCR analysis

Total RNA was extracted according to the Qiazol protocol (Qiagen Sciences, Maryland, USA). Reverse transcription was performed on 2 μg of total RNA using the high-capacity cDNA archive kit (Applied Biosystems). Real-time PCR was performed in an ABI Prism 7000 Sequence Detection System (Applied Biosystems) using TaqMan PCR Master Mix (Applied Biosystems) and the thermocycler parameters recommended by the manufacturer. PCRs were performed in duplicate in a total volume of 50 μl, containing 25 μl of the reverse transcription reaction. TaqMan gene expression assays were used for IL-2, IL-4, IFN-γ, leptin, ObR and TNF-α (Rn00587673_m1, Rn01456866_m1, Rn00594078_m1, Rn00565158_m1, Rn01433205_m1 and Rn01525859_g1, respectively; Applied Biosystems). All expression assays were performed following the manufacturer's procedures, except ObR, that was done following the modifications of Siegrist-Kaiser et al. [74]. Relative gene expression comparisons were carried-out using an invariant endogenous control (actin). According to manufacturer's guidelines, the ΔΔC_T_ method was used for relative quantification.

### Statistical analysis

Differences between groups were analyzed by a one-way ANOVA followed by a Bonferronís test. Statistical significance was set at p<0.05. Pearson's correlation coefficient r was used to measure the degree of association between different variables in each group. Two-tailed p values <0.05 were considered significant. These correlations were conducted with Prism software 4.00 (GraphPad, San Diego, CA, USA). The trapezoidal rule of GraphPad Prism was employed to calculate the area under the curve. Data are expressed as mean ± SEM.
